# The association between falls and depressive symptoms among older adults: evidence from the China Health and Retirement Longitudinal Study

**DOI:** 10.3389/fpubh.2023.1248551

**Published:** 2023-10-30

**Authors:** Zhiqiang Feng, Qi Chen, Yanjing Li, Zhen Xue, Xiaoning Hao

**Affiliations:** ^1^School of Economics, Peking University, Beijing, China; ^2^China National Health Development Research Center, Beijing, China

**Keywords:** depressive symptoms, falls, older adults, China, CART

## Abstract

**Background:**

Falls place a heavy burden on older adults and families, and there was little research on the relationship between falls and depressive symptoms among older adults in China. This study is designed to examine the association between falls and depressive symptoms in Chinese older adults.

**Methods:**

This study was based on 9,539 data sets from the China Health and Retirement Longitudinal Study (CHARLS) in 2018. The 10-item Center for Epidemiologic Studies-Depression Scale (CESD-10) was used to access depressive symptoms in older adults. A logistic regression model was used to calculate multivariate odds ratios (ORs) and 95% confidence intervals (CIs) for falls and depressive symptoms, adjusted for possible confounders. The Classification and regression tree (CART) demonstrates the prediction of the target variable values based on other variables.

**Results:**

In this study, 9,539 older people were selected: 60–69 years old accounted for 63.0%, 70–79 years old accounted for 29.7%, and 80 years old and above accounted for 7.3%. Male accounted for 49.7% and female for 50.3%. The rate of falls among older adults was 21.4%, and the rate of depressive symptoms was 33.9%. Adjusted ORs (OR = 1.37, 95% CI: 1.23, 1.53) showed a significant association between falls and depressive symptoms among older adults. Subgroup analysis revealed that this association was statistically significant across male (OR = 1.29, 95% CI: 1.23, 1.53) and female (OR = 1.42, 95% CI: 1.23, 1.64), 60–69 aged (OR = 1.38, 95% CI: 1.19, 1.60) and 70–79 aged (OR =1.42, 95% CI: 1.16, 1.74), rural (OR = 1.42, 95% CI: 1.25, 1.61), <15,000 CNY (OR = 1.35, 95% CI: 1.19, 1.54) and more than 25,000 CNY (OR = 1.42, 95% CI: 1.09, 1.85). Additionally, The CART model showed that the probability (73.0%) of falls was highest among older adults with depressive symptoms who self-rated poor health and female gender.

**Conclusions:**

This cross-sectional study demonstrated a significant association between falls and depressive symptoms in Chinese older adults. The findings provide some evidence and support for risk monitoring, screening for depressive symptoms, and early prevention in the high-risk older population.

## 1. Introduction

Falls are the leading cause of injury, disability and death among older adults, and are increasingly recognized as a severe public health problem among the older population (1, 2). It is estimated that nearly one-third of older adults have an accidental fall each year, and 50% of adults over 80 have an accidental fall (3, 4). Falls in older adults can lead to loss of function, including traumatic brain injury, hip fractures, and other moderate to severe injuries resulting in limited mobility, loss of autonomy, and death (5–7). As the most costly type of injury among older adults, falls place a heavy burden on individuals, their families, and society (8).

Depression is one of the most common mental health disorders in older adults, and it has become a severe health problem in both developed and developing countries around the world (9). Globally, 322 million people suffer from depression (10), accounting for 12.1% of total global disability life expectancy and 26.4% of total global disability-adjusted life expectancy (11). China has the largest older population in the world, and the challenge of depressive symptoms among older adults should be taken seriously enough.

Falls and depressive symptoms are both important older adult health issues. Although the findings were not entirely consistent, previous studies have shown that falls were associated with depressive symptoms, and the interaction of these two can lead to many health problems (12–14). In recent years, a series of studies in China have demonstrated that falls were associated with depressive symptoms using nationwide representative data from various perspectives, including age, gender, place of residence, and experience of falls (15–18). It should be noted that both depressive symptoms and falls are dynamic and undergo gradual change (19). It is significant to analyze and comprehend the current status, health characteristics, and association between falls and depressive symptoms among older Chinese adults to enrich the research in this area.

A better understanding of the relationship between falls and depressive symptoms will shed light on designing evidence-based prevention and interventions targeting this vulnerable population. We propose the hypothesis that there is an association between falls and depressive symptoms. We will draw profiles of falls and depressive symptoms among older adults in China using nationwide representative data from the China Health and Retirement Longitudinal Study (CHARLS). We will investigate the association between falls and depressive symptoms in older adults of different ages, gender, residence, and income through subgroup analyses while adjusting for various sociodemographic, lifestyle, disease, and health conditions. Subsequently, the Classification and regression tree (CART) demonstrates the prediction of the target variable values based on other variables. This study may expand our understanding of the relationship between falls and depressive symptoms in older adults in China and provide a valuable reference for related policy development.

## 2. Materials and methods

### 2.1. Design and study population

The data for this study were based on the third wave of the China Health and Retirement Longitudinal Study (CHARLS) conducted by Peking University in 2018, and the respondents of CHARLS were selected using a multistage stratified probability-proportional to size sampling (PPS) technique, which is a nationally representative community-based population survey (20). The project sampling covered 150 county-level units on the Chinese Mainland (scattered in 28 provincial units across the country), 450 village -level units, and about 10,000 households. A household survey was conducted among residents aged 45 and above. CHARLS carried out three national follow-up surveys in 2013, 2015, and 2018, of which 19,816 individual questionnaires for those aged 45 and over were successfully completed in 2018. The 2018 wave of CHARLS data is the most recent data available. In addition, the data is based on a national sample survey, which makes it a large and representative sample. Survey data and detailed information about the CHARLS can be accessed through its official website (charls.cer.edu.cn/en).

**Figure 1:**
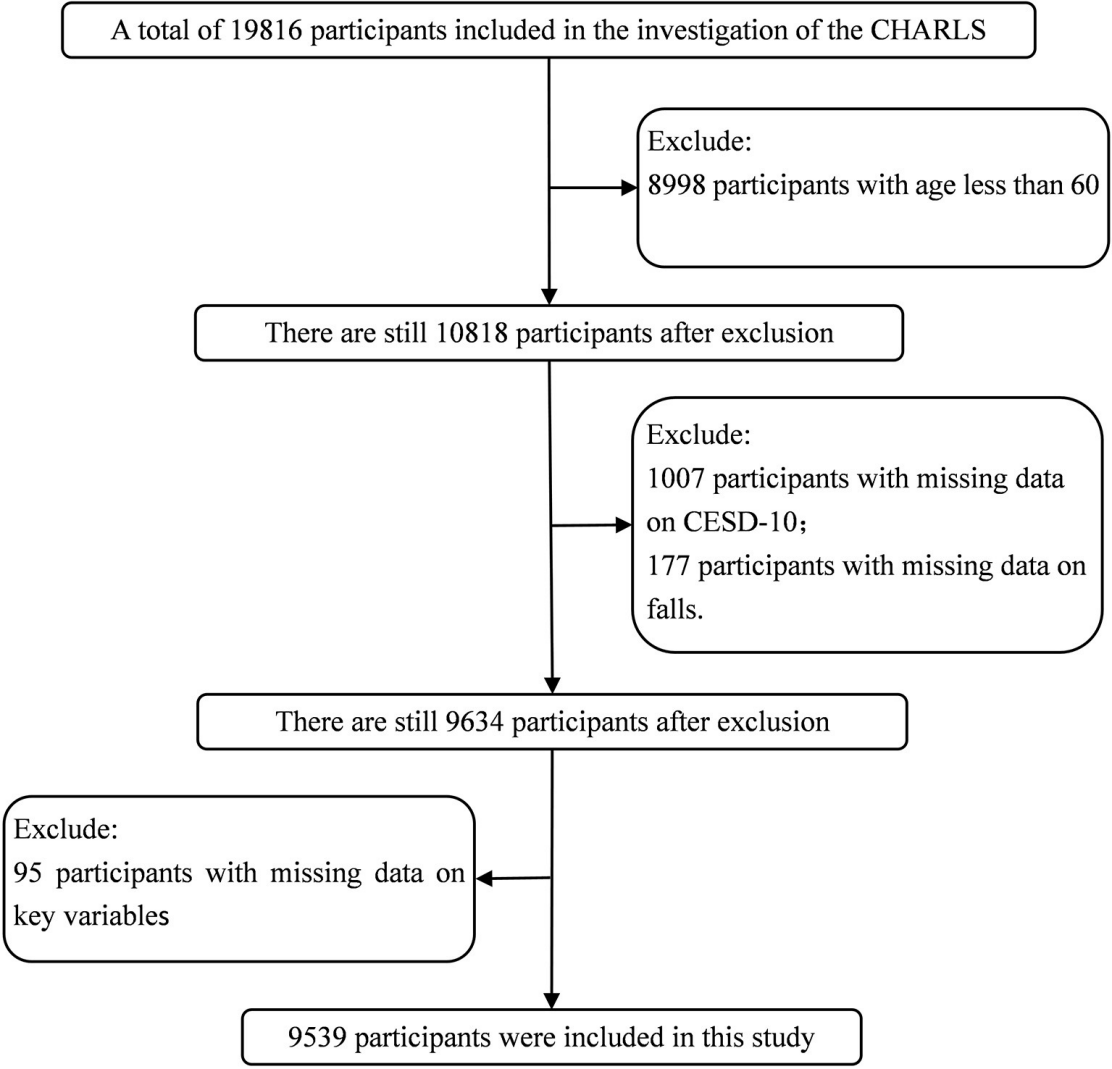
Flow chart of the selection process of participants.

Based on a large population-based study, the current study excluded participants if they were younger than 60 years old, missing CESD-10 date, missing falls date, and missing values in main variables. A total of 19,816 participants completed the CHARLS in 2018, 8,998 participants < 60 years old were excluded, 1,007 participants missing CESD-10 data were excluded, 177 participants were excluded for missing falls data, 95 participants missing values in main variables were excluded. After the above screening, 9,539 participants were finally included in our analysis (for the sample screening process, see Figure 1).

### 2.2. Depressive symptoms assessment

Our study measured depressive symptoms using the 10-item Center for Epidemiological Studies Depression Scale (CESD), the scale was presented in the first part of the Supplementary material. Survey respondents were asked about the number of days of relevant experience in the past week. Each item was rated on a four-point Likert scale: 0 (rarely or none of the time; < 1 day), 1 (some of the time; 1–2 days), 2 (much or a moderate amount of the time; 3–4 days), or 3 (most or all of the time; 5–7 days). The scale has a maximum score of 30 and a minimum score of 0, with higher scores indicating higher levels of depressive symptoms. A previous survey showed that the cut-off point of 10 had high levels of sensitivity (0.85) and specificity (0.80) in Chinese older adults (21), and the cut-off points of 10 used in CESD-10 have been validated in numerous studies of the older adults in China (22, 23). Thus, we used 10 as the cut-off point to generate the binary depression symptoms variable.

### 2.3. Falls assessment

Falls were evaluated based on the following single item, “Have you ever fallen during the past 2 years?” In this study, falls were dichotomized as no falls vs. ≥1 falls. Then we used a question to assess the condition of fall injury “how many times have you fallen down seriously enough to need medical treatment?”

### 2.4. Covariates

Our covariates included sex, age, marital status, education level, personal annual income (CNY), residence, health insurance, self-rated health, self-rated eyesight, medication use, smoking, drinking, number of Non-Communicable Diseases (NCDs), sleep time, Activities of Daily Living (ADL) and Instrumental Activities of Daily Living (IADL).

Sex was divided into male and female. Age was divided into three groups: 60–69, 70–79, ≥80. Marital status was divided into two groups: married, others (merged with single, divorced, widowed). Education level was divided into four groups: illiterate, primary, middle, college or higher (Primary: primary school and junior high school; Middle: high school, technical secondary school, vocational school; College or higher: some college, junior college, college or higher.). Personal annual income was categorized into 0–15,000, 15,001–25,000, and >25,000 CNY (equals to 0–2,268, 2,269–3,781, >3782 US dollars in 2018). The residence was divided into rural and urban. Health insurance was divided into two groups: yes, no.

Self-rated health and self-rated eyesight were divided into good, fair, and poor. Smoking and drinking were categorized as current, former, and never. Medication use included antidepressants or tranquilizers and sleeping pills divided into two groups: yes and no. The number of Non-Communicable Diseases for each respondent was categorized as none, 1, 2, and ≥3 (Chronic diseases including hypertension, dyslipidemia, diabetes, cancer, chronic lung diseases, liver or gallbladder disease, heart disease, stroke, kidney disease, stomach or other digestive diseases, emotional, nervous, or psychiatric problems, memory-related disease, rheumatism or arthritis, asthma). People with depressive symptoms have a high prevalence of sleep disorders, while most falls occur during sleep (24). In this study, sleep time was assessed based on one question: “During the past month, how many hours of actual sleep did you get at night (average hours for one night, this may be shorter than the number of hours you spend in bed.)?” The ADL included six activities: dressing, bathing/showering, eating, getting in and out of bed, using the toilet, and bladder and bowel control (25, 26). The IADL included six activities: doing household chores, preparing meals, shopping, making phone calls, managing money, and taking medications (27, 28). For both ADL and IADL, answers were categorized as a four-point Likert scale: 1 (do not have any difficulty), 2 (have difficulty but can still do it), 3 (have difficulty and need help), or 4 (can not do it). ADL and IADL scores ranged from 6 to 24, from lowest to highest dependency.

### 2.5. Data analysis

Descriptive statistic methods were used to analyze the participants' characteristics; Chi-square test and student's *t*-test were used to compare the difference in categorical and continuous variables. Then, multivariate logistic regression was used to assess the association between falls and depressive symptoms among older adults, and the results were expressed as adjusted odds ratios (OR-adjusted) and their 95% confidence intervals (95% CI).

A classification and regression tree model was used to explore further the interactions between falls and depressive symptoms in older adults. The CHAID (chi-squared automatic interaction detector) algorithm was selected for the filter variables for the metric setting of the classification and regression tree model. The CHAID algorithm decided to branch on the data based on the *p*-value in the node chi-square test, selecting the independent variable that interacted most strongly with the dependent variable at each step and merging the categories of each independent variable if they were not statistically different from the dependent variable. This analysis method can intuitively reflect the sample characteristics and the proportion of different results occurring and find the optimal classification variables and results by comparing numerous classification rules. It provided a reference basis for subsequent covariate mergers and divisions, reduced the influence of human subjective factors, and also reflected the existence of interactions between covariates (29–32). Decision tree models were pruned by setting the number of layers and minimum sample size of parent and child nodes. In this study, the maximum growth depth of the decision tree was set to three layers according to the research needs. In order to prevent the number of samples on a tree node from being too small, the minimum sample size on the parent node was specified to be 400 in advance, the minimum sample size on the child node was specified to be 200, and if the number of samples on the node did not meet this requirement, this node was the terminal node, and no further splitting was performed.

All statistical analyses were performed using SPSS version 26.0 (SPSS, Chicago, Illinois, USA). The level of significance was set at *P*-values < 0.05.

## 3. Results

### 3.1. Basic information of the participants

Table 1 provides the general characteristics of the participants in this study. Of the 9,539 participants, 3,235 had depressive symptoms, accounting for 33.9%. Generally speaking, the majority of older adults were female (50.3%), at the age of 60–69 (63.0%), married (80.3%), an illiterate education level (51.7%), personal annual income < 15,000 CNY (69.2%), residence in the rural (73.0%), with health insurance (97.2%), fair self-rated health (48.4%), bad self-rated eyesight (66.8%), never smoking (54.0%), never drinking (63.6%), no medication use (98.9%), no non-communicable chronic disease (52.9%), the average sleep time was 6.19 ± 2.05 h, ADL and IADL scores were 8.01 ± 2.70 and 7.58 ± 3.06, respectively. Among the participants, older adults were more likely to have depressive symptoms (all *P* < 0.05) if they were female (41.01%), marital status was other (41.44%), illiterate education level (40.91%), personal annual income < 15,000 CNY (38.33%), residence in rural areas (36.72%), without health insurance (40.98%), bad self-rated health (53.02%), bad self-rated eyesight (38.77%), never smoking (38.34%), former drinking (37.92%), used medication (83.7%), and had 3 or more chronic diseases (47.67%). Participants who were male (73.62%), married (67.93%), college or higher education level (88.18%), personal annual income more than 25,000 CNY (78.11%), residence in urban area (73.67%), with health insurance (66.29%), good self-rated health (81.96%), good self-rated eyesight (82.09%), current smoking (71.41%), current drinking (73.13%), no medication use (66.6%), no non-communicable chronic disease (71.29%) were less likely to have depressive symptoms (all *P* < 0.05). In addition, older adults with less sleep time (SD = 5.62 ± 2.17), lower ADL (SD = 7.85 ± 2.60), and higher IADL (8.61 ± 3.77) were more likely to have depressive symptoms (all *P* < 0.05).

**Table 1:** Comparison of sample characteristics between depressive and non-depressive among older adults.

**Characteristics**	***n* (%)**	**Depressive symptoms**		***P***
		**Yes (%)**	**No (%)**	
Observations	9,539	3,235 (33.9)	6,304 (66.1)	
**Sex**				**<0.001**
Male	4,738 (49.7)	1,250 (26.38)	3,488 (73.62)	
Female	4,801 (50.3)	1,958 (41.01)	2,816 (58.99)	
**Age**				0.632
60-	6,007 (63.0)	2,019 (33.61)	3,988 (66.39)	
70-	2,831 (29.7)	969 (34.23)	1,862 (65.77)	
80-	701 (7.3)	247 (35.24)	454 (64.76)	
**Marital status**				**<0.001**
Married	7,659 (80.3)	2,456 (32.07)	5,203 (67.93)	
Others^a^	1,880 (19.7)	779 (41.44)	1,101 (58.56)	
**Education** ^b^				**<0.001**
Illiterate	4,935 (51.7)	2,019 (40.91)	2,916 (59.09)	
Primary	3,655 (38.3)	1,032 (28.24)	2,623 (71.76)	
Middle	806 (8.4)	168 (20.84)	638 (79.16)	
College or higher	143 (1.5)	16 (11.19)	127 (88.81)	
**Annual income (CNY)**				
< 15,000	6,601 (69.2)	2,530 (38.33)	4,071 (61.67)	**<0.001**
15,000–25,000	795 (8.3)	236 (29.69)	559 (70.31)	
>25,000	2,143 (22.5)	469 (21.89)	1,674 (78.11)	
**Residence**				
Rural	6,964 (73.0)	2,557 (36.72)	4,407 (63.28)	**<0.001**
Urban	2,575 (27.0)	678 (26.33)	1,897 (73.67)	
**Health insurance**				
Yes	9,273 (97.2)	3,126 (33.71)	6,147 (66.29)	**0.015**
No	266 (2.8)	109 (40.98)	157 (59.02)	
**Self-rated health**				
Good	2,023 (21.2)	365 (18.04)	1,658 (81.96)	**<0.001**
Fair	4,621 (48.4)	1,335 (28.89)	3,286 (71.11)	
Poor	2,895 (30.3)	1,535 (53.02)	1,360 (46.98)	
**Self-rated eyesight**				**0.002**
Good	592 (6.2)	106 (17.91)	486 (82.09)	
Fair	2,579 (27.0)	660 (25.59)	1,919 (74.41)	
Poor	6,368 (66.8)	2,469 (38.77)	3,899 (61.23)	
**Smoking**				**<0.001**
Current	4,159 (43.6)	1,189 (28.59)	2,970 (71.41)	
Never	5,154 (54.0)	1,976 (38.34)	3,178 (61.66)	
Former	226 (2.4)	70 (30.97)	156 (69.03)	
**Drinking**				
Current	3,089 (32.4)	830 (26.87)	2,259 (73.13)	**<0.001**
Never	6,065 (63.6)	2,259 (37.25)	3,806 (62.75)	
Former	385 (4.0)	146 (37.92)	239 (62.08)	
**Medication use**				**0.03**
Yes	104 (1.1)	87 (83.7)	17 (16.3)	
No	9,435 (98.9)	3,148 (33.4)	6,287 (66.6)	
***NCDs*** ^c^				**0.007**
None	5,047 (52.9)	1,449 (28.71)	3,598 (71.29)	
1	2,712 (28.4)	995 (36.69)	1,717 (63.31)	
2	1,092 (11.4)	463 (42.40)	629 (57.60)	
≥3	688 (7.2)	328 (47.67)	360 (52.33)	
Sleep time-mean (SD)	6.19 ± 2.05	5.62 ± 2.17	6.48 ± 1.91	**0.003**
ADL-mean (SD)	8.01 ± 2.70	7.85 ± 2.60	8.10 ± 2.75	**0.004**
IADL-mean (SD)	7.58 ± 3.06	8.61 ± 3.77	7.05 ± 2.45	**0.002**

a Merged with single, divorced, widowed.

b Primary: primary school and junior high school; Middle: high school, technical secondary school, vocational school; College or higher: some college, junior college, college or higher.

c NCDs: non-communicable chronic diseases. Bold values represent *P* < 0.05 for statistical significance.

### 3.2. Prevalence of falls and medical treatment

Table 2 illustrates the prevalence of falls and medical treatment. About the 9,539 participants, 2,044 participants have experienced falls, accounting for 21.4%. The falls prevalence of participants with and without depressive symptoms was 32.8 and 15.6%, respectively. Among older adults who had experienced falls, 42.9% had been medically treated for severe fall, with the percentages of 1 time, 2 times, and ≥3 times was 33.9, 5.3, and 3.7%, respectively.

**Table 2:** Prevalence of falls and medical treatment.

**Characteristics**	**Total, *n***	**Yes, *n***	**%**
**Falls**	9,539	2,044	21.4
With depressive symptoms	3,235	1,062	32.8
Without depressive symptoms	6,304	982	15.6
**Times of medical treatment for severe falls**	2,044	877	42.9
0 times		1,167	57.1
1 times		693	33.9
2 times		109	5.3
≥3 times		75	3.7

**Figure 2:**
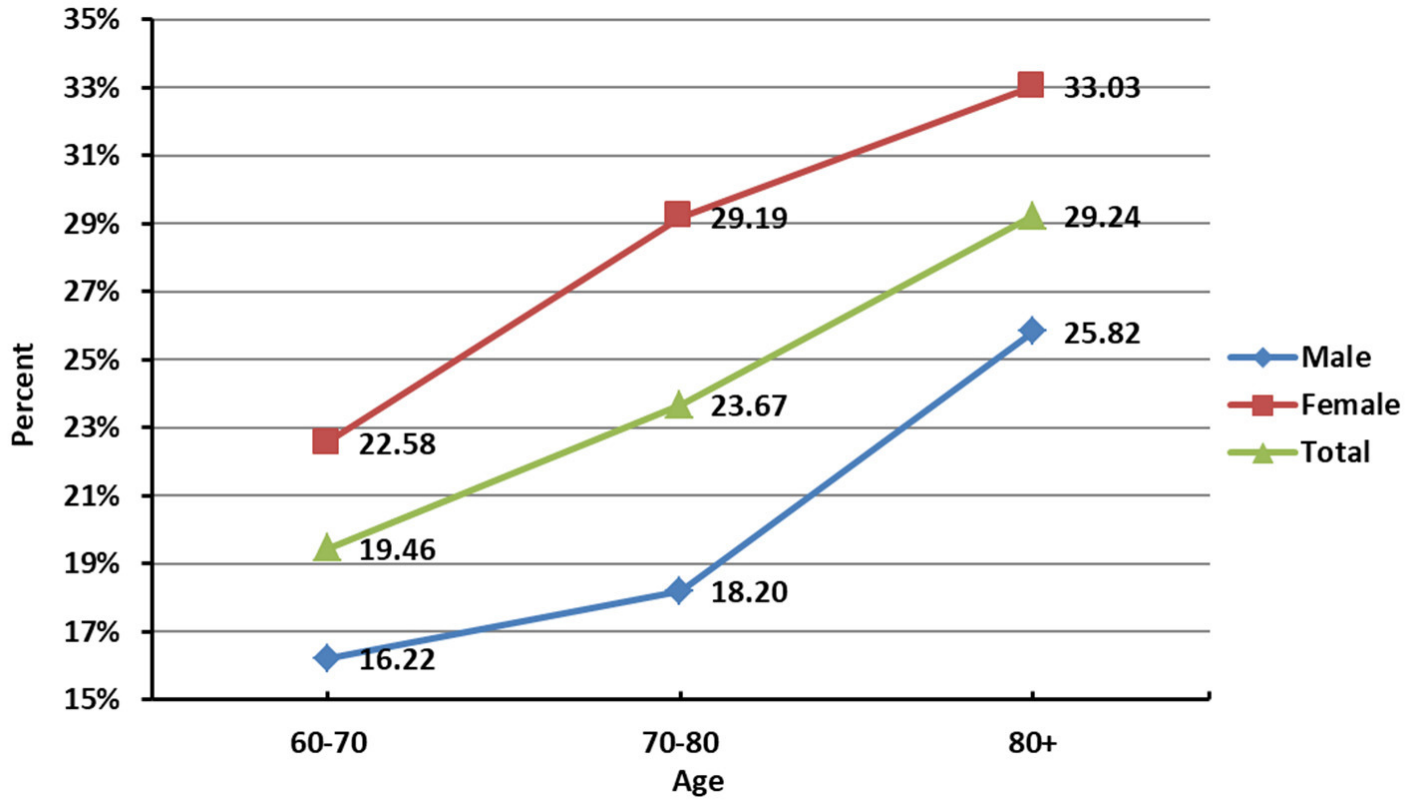
Fall rate of older adults in different age groups.

We found that older adults with depressive symptoms have higher rates of falls than those without depressive symptoms, the difference was statistically significant (*P* < 0.001). Further, the association between depressive symptoms and falls in older adults of different ages and genders subgroups was analyzed (Table 3), and significant associations were found in male at age 60- (OR = 1.74, 95% CI: 1.41, 2.16), 70- (OR = 1.57, 95% CI: 1.16, 2.11), and female at age 60- (OR = 1.78, 95% CI: 1.49, 2.13), 70- (OR = 1.83, 95% CI: 1.44, 2.33). As shown in Figure 2 increases with age, and the fall rate of females was higher than that of males in the same age group.

**Table 3:** Association between depression and falls in older adults by gender and age subgroups.

**Age subgroups**	**Male (*****n*** = **4,738)**		**Female (*****n*** = **4,801)**	
	**Unadjusted OR (95% CI)**	**Adjusted OR (95% CI)^a^**	**Unadjusted OR (95% CI)**	**Adjusted OR (95% CI)^a^**
60- (*n* = 6,007)	1.89 (1.53, 2.32)^***^	1.74(1.41, 2.16)^***^	1.91 (1.61, 2.27)^***^	1.78 (1.49, 2.13)^***^
70- (*n* = 2,831)	1.69 (1.27, 2.26)^***^	1.57 (1.16, 2.11)^**^	1.98 (1.57, 2.50)^***^	1.83 (1.44, 2.33)^***^
80- (*n* = 701)	1.10 (0.66,1.85)	1.05 (0.61, 1.79)	1.68 (1.06, 2.67)^*^	1.51 (0.93, 2.45)

a Adjusted OR: Adjusted for marital status, education level, personal annual income, residence, health insurance, NCDs.

OR, odds ratio; CI, confidence interval.

* *P* < 0.05;

** *P* < 0.01,

*** *P* < 0.001.

### 3.3. Falls and depressive symptoms

In Table 4, we used three sets of logistic regression models (unadjusted model, model 1, and model 2) to examine the associations between falls and depressive symptoms, more detailed results of the logistic regression analysis were presented in the second part of the Supplementary material. After being adjusted with gender, age, marital status, education level, personal annual income, residence, and health insurance (model 1), the result shows that falls (OR = 1.80, 95% CI: 1.62, 2.00) were associated with depressive symptoms. In Model 2, although the OR value (OR = 1.37, 95% CI: 1.23, 1.53) becomes lower after being adjusted with all of the covariates, the existence of the association between falls and depressive symptoms can still be shown.

**Table 4:** Logistic regression analysis of falls and depressive symptoms among subgroups.

**Characteristics**	**Unadjusted model^a^**	**Model 1^b^**	**Model 2^c^**
	**OR (95% CI)**	**OR (95% CI)**	**OR (95% CI)**
All participants (*n* = 9,539)	1.95 (1.76, 2.15)	1.80 (1.62, 2.00)	1.37 (1.23, 1.53)
	***P* < 0.001**	***P* < 0.001**	***P* < 0.001**
**Gender subgroups**			
Male (*n* = 4,738)	1.73 (1.48, 2.03)	1.72 (1.46, 2.02)	1.29 (1.23, 1.53)
	***P* < 0.001**	***P* < 0.001**	***P* = 0.004**
Female (*n* = 4,801)	1.92 (1.68, 2.19)	1.84 (1.61, 2.11)	1.42 (1.23, 1.64)
	***P* < 0.001**	***P* < 0.001**	***P* < 0.001**
**Age subgroups**			
60- (*n* = 6,007)	1.99 (1.75, 2.27)	1.86 (1.63, 2.13)	1.38 (1.19, 1.60)
	***P* < 0.001**	***P* < 0.001**	***P* < 0.001**
70- (*n* = 2,831)	2.03 (1.70, 2.42)	1.82 (1.51, 2.18)	1.42 (1.16, 1.74)
	***P* < 0.001**	***P* < 0.001**	***P* < 0.001**
80- (*n* = 701)	1.46 (1.04, 2.04)	1.38 (0.98, 1.94)	1.05 (0.72, 1.53)
	***P* = 0.027**	*P* = 0.067	*P* = 0.806
**Residence subgroups**			
Rural (*n* = 6,964)	1.99 (1.78, 2.23)	1.84 (1.63, 2.07)	1.42 (1.25, 1.61)
	***P* < 0.001**	***P* < 0.001**	***P* < 0.001**
Urban (*n* = 2,575)	1.71 (1.39, 2.12)	1.68 (1.35, 2.09)	1.22 (0.96, 1.56)
	***P* < 0.001**	***P* < 0.001**	*P* = 0.104
**Annual income subgroups**			
< 15,000 (*n* = 6,601)	1.94 (1.73, 2.18)	1.80 (1.60, 2.03)	1.35 (1.19, 1.54)
	***P* < 0.001**	***P* < 0.001**	***P* < 0.001**
15,000–25,000 (*n* = 795)	1.80 (1.23, 2.65)	1.74 (1.17, 2.59)	1.52 (0.985, 2.36)
	***P* = 0.003**	***P* = 0.006**	*P* = 0.059
>25,000 (*n* = 2,143)	1.87 (1.47, 2.38)	1.83 (1.43, 2.34)	1.42 (1.09, 1.85)
	***P* < 0.001**	***P* < 0.001**	***P* = 0.009**

a Unadjusted model: falls alone.

b Model 1: controls for gender, age, marital status, education level, personal annual income, residence, health insurance.

c Model 2: controls for Model 1 covariates, as well as self-rated health, self-rated eyesight, smoking, drinking, medication use, NCDs, sleep time, ADL, and IADL.

OR, odds ratio; CI, confidence interval. Bold values represent *P* < 0.05 for statistical significance.

Regarding the subgroups divided according to gender, significant associations were found for both male (OR = 1.29, 95% CI: 1.23, 1.53) and female (OR = 1.42, 95% CI: 1.23, 1.64) subgroups. Moreover, the ORs of female subgroup were higher than that of the male in all models; The OR value of the age subgroup of the 70–79 (OR = 1.42, 95% CI: 1.16, 1.74) people was higher than that of the 60–69 (OR = 1.38, 95% CI: 1.19, 1.60) people in all models, but the OR value in ≥80 people did not show a significant association; Also, the OR values of the subgroups by place of residence showed that only the rural (OR = 1.42, 95% CI: 1.25, 1.61) had statistically significant ORs; In the subgroup of annual income, personal annual income < 15,000 CNY (OR = 1.35, 95% CI: 1.19, 1.54) and more than 25,000 CNY (OR = 1.42, 95% CI: 1.09, 1.85) showed a statistically significant association.

### 3.4. CART model analysis

**Figure 3:**
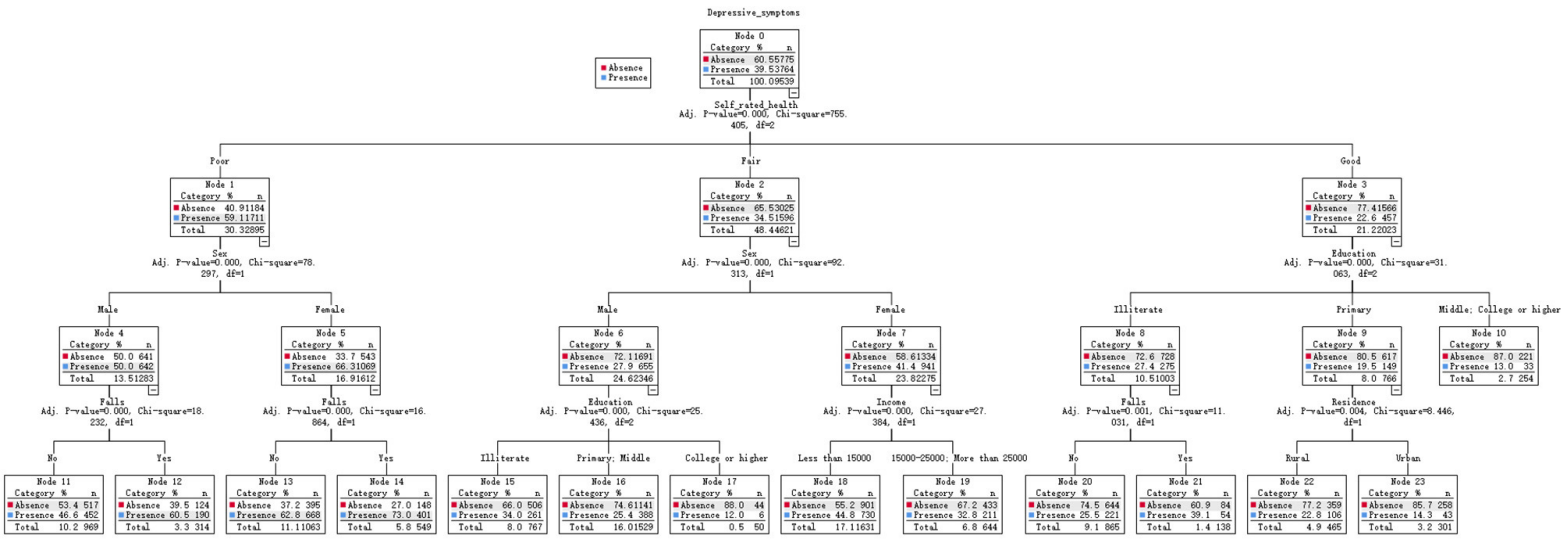
Regression tree model of depression and falls in older adults.

As shown in Figure 3, the interaction between falls and depressive symptoms in older adults was analyzed using the CATR model, which contains 3 depths, 23 nodes, and 12 terminal nodes, the *p*-values of the chi-square test for all nodes met the criteria (*P* < 0.001). The CART model analysis found that depressive symptoms in older adults were mainly related to falls, gender, education, annual income, residence, and self-rated health. In this model, self-rated health was the first split factor, the second level was split at gender and education, and the end nodes of the model were falls, education, income, and residence. For those older adults with depressive symptoms, self-rated good health (node 3) and degree of illiterate (node 8), the possibility (39.1%) of falls (node 21) was lowest. Conversely, for those with poor self-rated health (node 1), female gender (node 5), the possibility (73.0%) of falls (node 14) was the highest.

## 4. Discussion

This study aimed to explicitly investigate the association between falls and depressive symptoms in the older adults in China while focusing on whether an interaction exists between falls, depressive symptoms, and demographic characteristics based on the nationally representative sample of the older adults in China. When we adjusted for possible confounders, the associations between falls and depressive symptoms remained, although the strength of the association was reduced. Further subgroup analysis found statistically significant associations between falls and depressive symptoms among males, female, 60–69 aged, 70–79 aged, <15,000 CNY, and more than 25,000 CNY in the personal annual income subgroups.

This study found that the prevalence of falls in older adults was 21.4%, lower than those found in other studies focusing on other countries' older adults, such as 31% in the United States (33), 25.6% in Australia (34), 20–30% in Canada (35) and 27.6% in Brazil (36). This difference may be related to the age grouping and the time frame of the measurement. We also found that the rate of falls in older adults increased with age, with females having a higher rate of falls than males in the same age group, consistent with previous research findings (37, 38). The prevalence of depressive symptoms among older adults in this study was 33.9%, which was higher than that in the United States (23.97%) (4), South Korea (16.08%) (5), and Singapore (7.8%) (6). Due to different cultural backgrounds, environment and application measures, and methodological differences (including the screening scale used and the cut-off point adopted), differences in depressive symptoms between countries should be compared with caution.

Several studies have analyzed the relationship between falls and depressive symptoms, and our findings further confirm this result (39–41). A cross-sectional analysis of 1,261 community-dwelling older adults showed a significant association between depressive symptoms and falls (42). In several prospective studies, depressive symptoms have been identified as a risk factor for falls (14, 43, 44). A meta-analysis of 20 prospective studies found that depressive symptoms in older adults were a risk factor for falls, regardless of how depressive symptoms and falls were measured, as well as different follow-up times and statistical methods (45). A 2-year follow-up cohort study showed that depressive symptoms significantly increased the risk of unexplained falls and the association with accidental falls was only close to borderline significance (12). Despite this, the mechanism for this relationship remains unclear. One possible mechanism is that depressive symptoms are often associated with greater physical impairment and cognitive deficits (46, 47). Another possible mechanism is that antidepressants affect attention, gait, balance, and blood pressure regulation (48). Besides, fear of falling is another critical factor in the case of older adults. Fear of falling has been related to anxiety and symptoms of depression, which may mediate the relationship between physical and cognitive factors, depressive symptoms, and falls (49, 50).

In our study, we found a significant association between depressive symptoms and falls among the Chinese older adults population, as well as specific subgroups. This finding may indicated a potential association between falls and depressive symptoms among the older adults. Subgroup analysis showed an association between falls and depressive symptoms in 60–69, 70–79 aged, while this association was not significant in those over 80 aged. One possible reason for this is the reduced mobility and physical activity of older adults with increasing age, reducing the chances of falls (51–53). Interestingly, after controlling for health-related factors, subgroups by place of residence showed no statistically significant association for urban older adults, with only those living in rural areas confirming the association. In China, medical conditions, economic conditions and living facilities in rural areas are inferior to those in urban areas. Compared to rural older adults, urban older adults have access to high-quality and timely preventive care and medical services when needed, and these differences may have a direct or indirect effect on falls and depressive symptoms in older adults (54–56). Besides, in a subgroup analysis of income, we found a statistically significant relationship between falls and depressive symptoms in older adults with annual incomes < 15,000 CNY and >25,000 CNY, while the relationship approached borderline significance for those in the 15,000–25,000 CNY income subgroup. It has been argued that financial income makes older adults feel more secure in their financial independence, reduces poverty-related stress, and trades off health-generating resources such as health care and better housing conditions (57, 58). More and more evidence suggests that older adults with low socioeconomic status are vulnerable to developing falls and depressive symptoms (59, 60). Since cross-sectional data were used, our analysis cannot examine causality and more research needs to be conducted further to analyze the relationship between income levels and them.

Our study investigated the association between falls and depressive symptoms and related demographic characteristics in an older Chinese population based on a national sample. The CART model was used to explore further the interactions between falls and depressive symptoms and its demographic variables. This study found that older adults with depressive symptoms, self-rated good health and degree of illiterate, the possibility of falls was lowest. Meanwhile, for those with poor self-rated health, female gender, the possibility of falls was the highest. The CART model can analyze non-linear and highly correlated data without pre-processing, it can visualize the importance of early warning indicators and their interactions, which is represented in the form of a tree diagram. By using the CART model, we conducted a more in-depth analysis of the data results on the relationship between depressive symptoms and falls in older adults, identifying people at risk for different characteristics by percentage. This study further explored the relationship between falls and depressive symptoms in older adults using data from a large sample. Further subgroup analyses found that the association between falls and depressive symptoms did not hold for older adults over 80 years and residents in urban after controlling for relevant covariates. The CART analysis found that older adults with depressive symptoms, poor self-rated health, and females had the highest probability of falling. This study enriched related studies and have some clinical and research implications. Furthermore, the findings related to falls and depressive symptoms in older adults may provide some rationale and support for implementing measures such as risk monitoring, screening for depressive symptoms, and early prevention in high-risk older adult populations.

This study has several limitations as follows. Firstly, study data were obtained through participant self-reports, which are inevitably biased. Secondly, this study is an analysis based on cross-sectional data, and we can only demonstrate an association between falls and depressive symptoms in older adults. The conclusion of causality needs to be proven by further studies. Thirdly, we control for relevant covariates in our analysis, but there is still a portion of unknown factors that we cannot exclude. Although we adjusted for the use of the medication, information regarding the dose, dosing schedule, adherence and start of treatment was not available, which may have led to some bias.

## 5. Conclusion

This cross-sectional study provides evidence of the significant association between falls and depressive symptoms in older Chinese adults, and subgroup analyses further demonstrated differences in the relationship across age, residence, and income groups. The probability of falls was highest among older adults with depressive symptoms who self-rated poor health and female gender. The findings provide some evidence and support for risk monitoring, screening for depressive symptoms, and early prevention in the high-risk older population.

## Data availability statement

Publicly available datasets were analyzed in this study. This data can be found here: https://charls.charlsdata.com/pages/Data/2018-charls-wave4/en.html.

## Ethics statement

Written informed consent was obtained from the individual(s) for the publication of any potentially identifiable images or data included in this article.

## Author contributions

ZF and XH: conceptualization, methodology, and writing—review and editing. ZF and QC: software, formal analysis, resources, data curation, and writing—original draft preparation. ZF, YL, and ZX: validation and investigation. XH: supervision, project administration, and funding acquisition. All authors have read and agreed to the published version of the manuscript.
