# Supplementary material for: The association between falls and depressive symptoms among older adults: evidence from the China Health and Retirement Longitudinal Study
Source: Front Public Health. 2023 Oct 30;11:1248551. doi: 10.3389/fpubh.2023.1248551 (PMC10643149; doi:10.3389/fpubh.2023.1248551)
Supplement: Supplementary file 1 [file Data_Sheet_1.pdf]

Supplementary Material

The association between falls and depressive symptoms among older adults: evidence from the China Health and Retirement Longitudinal Study (CHARLS)

Zhiqiang Feng [1, 2], Qi Chen [2], Yanjing Li [2], Zhen Xue [2], Xiaoning Hao [2] *

[1] School of Economics, Peking University, Beijing 100871, China

[2] National Health Development Research Center, Beijing 100191, China

*Corresponding author: Dr. Hao Xiaoning, China National Health Development Research Center, Beijing 100191, China. E-mail: xnhao5421@163.com

*** Correspondence:**

Corresponding author: Dr. Hao Xiaoning, China National Health Development Research Center, Beijing 100191, China. E-mail: xnhao5421@163.com

① CES-D questions, English and Mandarin.

1. I was bothered by things that don't usually bother me. 我因一些小事而烦恼。

(1) Rarely or none of the time (<1 day) 很少或者根本没有（<1天）
(2) Some or a little of the time (1-2 days) 不太多 （1-2天）
(3) Occasionally or a moderate amount of the time (3-4 days) 有时或者说有一半的时间（3-4天）
(4) Most or all of the time (5-7 days) 大多数的时间(5-7天)

2. I had trouble keeping my mind on what I was doing. 我在做事时很难集中精力。

(1) Rarely or none of the time (<1 day) 很少或者根本没有（< 1 天）
(2) Some or a little of the time (1-2 days) 不太多（ 1 - 2 天）
(3) Occasionally or a moderate amount of the time (3-4 days) 有时或者说有一半的时间（3-4天）
(4) Most or all of the time (5-7 days) 大多数的时间（5-7天）

3. I felt depressed.我感到情绪低落。

(1) Rarely or none of the time (<1 day) 很少或者根本没有（< 1 天）
(2)Some or a little of the time (1-2 days) 不太多（ 1 - 2 天）
(3)Occasionally or a moderate amount of the time (3-4 days) 有时或者说有一半的时间（3-4天）

(4)Most or all of the time (5-7 days) 大多数的时间（5-7天）

4. I felt everything I did was an effort.我觉得做任何事都很费劲。

(1) Rarely or none of the time (<1 day) 很少或者根本没有（< 1 天）
(2) Some or a little of the time (1-2 days) 不太多（ 1 - 2 天）
(3) Occasionally or a moderate amount of the time (3-4 days) 有时或者说有一半的时间（3-4天）
(4) Most or all of the time (5-7 days) 大多数的时间（5-7天）

5. I felt hopeful about the future. 我对未来充满希望。

(1) Rarely or none of the time (<1 day) 很少或者根本没有（< 1 天）
(2) Some or a little of the time (1-2 days) 不太多（ 1 - 2 天）
(3) Occasionally or a moderate amount of the time (3-4 days) 有时或者说有一半的时间（3-4天）
(4) Most or all of the time (5-7 days) 大多数的时间（5-7天）

6. I felt fearful. 我感到害怕。

(1) Rarely or none of the time (<1 day) 很少或者根本没有（< 1 天）
(2) Some or a little of the time (1-2 days) 不太多（ 1 - 2 天）
(3) Occasionally or a moderate amount of the time (3-4 days) 有时或者说有一半的时间（3-4天）
(4) Most or all of the time (5-7 days) 大多数的时间（5-7天）

7.My sleep was restless.我的睡眠不好。

(1) Rarely or none of the time (<1 day) 很少或者根本没有（< 1 天）
(2) Some or a little of the time (1-2 days) 不太多（ 1 - 2 天）
(3) Occasionally or a moderate amount of the time (3-4 days) 有时或者说有一半的时间（3-4天）
(4) Most or all of the time (5-7 days) 大多数的时间（5-7天）

8. I was happy. 我很愉快。

(1) Rarely or none of the time (<1 day) 很少或者根本没有（< 1 天）
(2) Some or a little of the time (1-2 days) 不太多（ 1 - 2 天）
(3) Occasionally or a moderate amount of the time (3-4 days) 有时或者说有一半的时间（3-4天）
(4) Most or all of the time (5-7 days) 大多数的时间（5-7天）

9. I felt lonely. 我感到孤独。

(1) Rarely or none of the time (<1 day) 很少或者根本没有（< 1 天）
(2) Some or a little of the time (1-2 days) 不太多（ 1 - 2 天）
(3) Occasionally or a moderate amount of the time (3-4 days) 有时或者说有一半的时间（3-4天）
(4) Most or all of the time (5-7 days) 大多数的时间（5-7天）

10. I could not get "going." 我觉得我无法继续我的生活。

(1) Rarely or none of the time (<1 day) 很少或者根本没有（< 1 天）
(2) Some or a little of the time (1-2 days) 不太多（ 1 - 2 天）

(3) Occasionally or a moderate amount of the time (3-4 days) 有时或者说有一半的时间（3-4天）

(4) Most or all of the time (5-7 days) 大多数的时间（5-7天）

②Logistic regression analysis of falls and depressive symptoms among subgroups

Table 1. Association between falls and depressive symptoms among older adults in China, CLHLS 2018 (n = 9539).

Characteristics	Unadjusted model	Model 1	Model 2
	OR (95% CI)	OR (95% CI)	OR (95% CI)
**Falls**		l	
No	1.0	1.0	1.0
Yes	1.95 (1.76, 2.15) ***	1.80 (1.62, 2.00) ***	1.37 (1.23, 1.53) ***
**Sex**			
Female		1.0	1.0
Male		0.63 (0.57, 0.69) ***	0.71 (0.61, 0.82) ***
**Age**			
60-		1.0	1.0
70--		0.97 (0.88, 1.07)	0.82 (0.73, 0.91) ***
80-		0.94 (0.79, 1.13)	0.70 (0.57, 0.86) **
**Marital status**			
Others		1.0	1.0
Married		0.81 (0.72, 0.90) ***	0.82 (0.73, 0.91) ***
**Education**			
Illiterate		1.0	1.0
Primary		0.76 (0.69, 0.84) ***	0.82 (0.73, 0.91) ***
Middle		0.60 (0.50, 0.73) ***	0.70 (0.57, 0.86) **
College or higher		0.35 (0.20, 0.60) ***	0.41 (0.23, 0.71) **
**Annual income (CNY)**			
＜15000		1.0	1.0
15000-25000		0.80 (0.68, 0.95) **	0.89 (0.74, 1.06)
＞25000		0.64 (0.56, 0.72) ***	0.71 (0.62, 0.82) ***
**Residence**			
Rural		1.0	1.0
Urban		0.84 (0.75, 0.94) **	0.86 (0.76, 0.98) *
**Health insurance**			
No		1.0	1.0
Yes		0.94 (0.73, 1.22)	0.90 (0.68, 1.18)
**Self-rated health**			
Good			1.0
Fair			0.54 (0.49, 0.61) ***
Bad			0.39 (0.33, 0.45) ***
**Self-rated eyesight**			
Good			1.0
Fair			0.76 (0.67, 0.85) ***
Bad			0.67 (0.53, 0.84) **
**Smoking**			
Current			1.0
Never			1.00 (0.87, 1.16)
Former			1.07 (1.78, 1.47)
**Drinking**			
Current			1.0
Never			0.99 (0.88, 1.11)
Former			1.17 (0.91, 1.49)
**Medication use**			
No			1.0
Yes			3.14 (2.69, 4.14) ***

**NCDs**	
None	1.0
1	1.17 (1.05, 1.30) **
2	1.34 (1.15, 1.55) ***
≥3	1.51 (1.26, 1.81) ***
**Sleep time-mean (sd)**	0.86 (0.84, 0.88) ***
**ADL-mean (sd)**	0.97 (0.95, 0.99) **
**IADL-mean (sd)**	1.10 (1.09, 1.12) ***

*P<0.05, **P<0.01, ***P<0.001

OR: odds ratio, CI: confidence interval

Unadjusted model: fall accidents alone

Model 1: controls for gender, age, marital status, education level, personal annual income, residence, health insurance

Model 2: controls for Model 1 covariates, as well as self-rated health, self-rated eyesight, smoking, drinking, medication use, NCDs, sleep time, ADL, and IADL.

Table 2. Association between falls and depressive symptoms among male older adults in China, CLHLS 2018 (n = 4738)

Characteristics	Unadjusted model	Model 1	Model 2
	OR (95% CI)	OR (95% CI)	OR (95% CI)
**Falls**			
No	1.0	1.0	1.0
Yes	1.73 (1.48, 2.03) ***	1.72 (1.46, 2.02) ***	1.29 (1.23, 1.53) **
**Age**			
60-		1.0	1.0
70--		0.98 (0.84, 1.13)	0.84 (0.72, 0.99) *
80-		1.00 (0.78, 1.29)	0.82 (0.62, 1.08)
**Marital status**			
Others		1.0	1.0
Married		0.93 (0.76, 1.13)	0.94 (0.76, 1.16)
**Education**			
Illiterate		1.0	1.0
Primary		0.68 (0.59, 0.79) ***	0.73 (0.62, 0.85) ***
Middle		0.67 (0.53, 0.85) **	0.78 (0.61, 1.01)
College or higher		0.33 (0.17, 0.62) **	0.38 (0.19, 0.75) **
**Annual income (CNY)**			
＜15000		1.0	1.0
15000-25000		0.79 (0.62, 1.02)	0.92 (0.71, 1.20)
＞25000		0.61 (0.62, 1.02) ***	0.71 (0.59, 0.86) ***
**Residence**			
Rural		1.0	1.0
Urban		0.96 (0.81, 1.15)	0.86 (0.76, 0.98) *
**Health insurance**			
No		1.0	1.0
Yes		0.98 (0.63, 1.53)	0.92 (0.76, 1.11)
**Self-rated health**			
Good			1.0
Fair			0.58 (0.49, 0.68) ***
Bad			0.43 (0.34, 0.54) ***
**Self-rated eyesight**			
Good			1.0
Fair			0.75 (0.63, 0.88) **
Bad			0.67 (0.49, 0.93) *
**Smoking**			
Current			1.0
Never			1.06 (0.88, 1.28)
Former			1.06 (0.75, 1.51)
**Drinking**			
Current			1.0
Never			1.07 (0.92, 1.24)
Former			1.17 (0.88, 1.55)
**Medication use**			
No			1.0
Yes			3.36 (2.29, 5.46) ***
**NCDs**			
None			1.0
1			1.18 (1.00, 1.39) *
2			1.36 (1.09, 1.70) **
≥3			1.50 (1.15, 1.96) **

**Sleep time-mean (sd)**	0.85 (0.82, 0.88) ***
**ADL-mean (sd)**	0.96 (0.93, 0.98) **
**IADL-mean (sd)**	1.10 (1.07, 1.12) ***

**P*<0.05, ***P*<0.01, ****P*<0.001
OR: odds ratio, CI: confidence interval
Unadjusted model: fall accidents alone
Model 1: controls for gender, age, marital status, education level, personal annual income, residence, health insurance
Model 2: controls for Model 1 covariates, as well as self-rated health, self-rated eyesight, smoking, drinking, medication use, NCDs, sleep time, ADL, and IADL.

Table 3. Association between falls and depressive symptoms among female older adults in China, CLHLS 2018 (n = 4801)

Characteristics	Unadjusted model	Model 1	Model 2
	OR (95% CI)	OR (95% CI)	OR (95% CI)
**Falls**			
No	1.0	1.0	1.0
Yes	1.92 (1.68, 2.19) ***	1.84 (1.61, 2.11) ***	1.42 (1.23, 1.64) ***
**Age**			
60-		1.0	1.0
70--		0.95 (0.83, 1.09)	0.77 (0.66, 0.89) ***
80-		0.89 (0.69, 1.14)	0.70 (0.53, 0.93) **
**Marital status**			
Others		1.0	1.0
Married		0.75 (0.65, 0.86) ***	0.77 (0.66, 0.89) **
**Education**			
Illiterate		1.0	1.0
Primary		0.86 (0.75, 1.00) *	0.94 (0.81, 1.10)
Middle		0.46 (0.33, 0.64) ***	0.53 (0.37, 0.75) ***
College or higher		0.34 (0.13, 0.90) *	0.43 (0.16, 1.17) **
**Annual income (CNY)**			
＜15000		1.0	1.0
15000-25000		0.82 (0.65, 1.02)	0.86 (0.67, 1.09)
＞25000		0.65 (0.54, 0.79) ***	0.71 (0.58, 0.87) **
**Residence**			
Rural		1.0	1.0
Urban		0.76 (0.65, 0.88) ***	0.82 (0.70, 0.97) *
**Health insurance**			
No		1.0	1.0
Yes		0.92 (0.67, 1.27)	0.95 (0.68, 1.35)
**Self-rated health**			
Good			1.0
Fair			0.52 (0.45, 0.61) ***
Bad			0.36 (0.29, 0.44) ***
**Self-rated eyesight**			
Good			1.0
Fair			0.76 (0.65, 0.88) ***
Bad			0.65 (0.45, 0.92) *
**Smoking**			
Current			1.0
Never			0.93 (0.75, 1.15)
Former			1.00 (0.48, 2.09)
**Drinking**			
Current			1.0
Never			0.9 (0.74, 1.08)
Former			1.27 (0.76, 2.12)
**Medication use**			
No			1.0
Yes			3.14 (2.24, 4.73) ***
**NCDs**			
None			1.0
1			1.16 (1.00, 1.34) *
2			1.34 (1.10, 1.64) **
≥3			1.52 (1.19, 1.96) **

**Sleep time-mean (sd)**	0.86 (0.84, 0.89) ***
**ADL-mean (sd)**	0.99 (0.96, 1.01)
**IADL-mean (sd)**	1.11 (1.09, 1.14) ***

**P*<0.05, ***P*<0.01, ****P*<0.001

OR: odds ratio, CI: confidence interval

Unadjusted model: fall accidents alone

Model 1: controls for gender, age, marital status, education level, personal annual income, residence, health insurance

Model 2: controls for Model 1 covariates, as well as self-rated health, self-rated eyesight, smoking, drinking, medication use, NCDs, sleep time, ADL, and IADL.

Table 4. Association between falls and depressive symptoms among 60-69 aged older adults in China, CLHLS 2018 (n = 6007)

Characteristics	Unadjusted model	Model 1	Model 2
	OR (95% CI)	OR (95% CI)	OR (95% CI)
**Falls**			
No	1.0	1.0	1.0
Yes	1.99 (1.75, 2.27) ***	1.86 (1.63, 2.13) ***	1.38 (1.19, 1.60) ***
**Sex**			
Female		1.0	1.0
Male		0.64 (0.57, 0.72) ***	0.68 (0.56, 0.83) **
**Marital status**			
Others		1.0	1.0
Married		0.78 (0.66, 0.92) **	0.79 (0.66, 0.94)
**Education**			
Illiterate		1.0	1.0
Primary		0.72 (0.63, 0.82) ***	0.79 (0.69, 0.91) **
Middle		0.60 (0.48, 0.75) ***	0.72 (0.57, 0.91) **
College or higher		0.26 (0.10, 0.66) **	0.31 (0.12, 0.82) *
**Annual income (CNY)**			
＜15000		1.0	1.0
15000-25000		0.78 (0.64, 0.96) *	0.9 (0.72, 1.11)
＞25000		0.63 (0.53, 0.74) ***	0.74 (0.62, 0.88) **
**Residence**			
Rural		1.0	1.0
Urban		0.83 (0.72, 0.96) *	0.83 (0.71, 0.96) *
**Health insurance**			
No		1.0	1.0
Yes		0.95 (0.65, 1.38)	0.96 (0.64, 1.44)
**Self-rated health**			
Good			1.0
Fair			0.53 (0.46, 0.61) ***
Bad			0.41 (0.34, 0.50) ***
**Self-rated eyesight**			
Good			1.0
Fair			0.76 (0.66, 0.88) ***
Bad			0.58 (0.43, 0.78) ***
**Smoking**			
Current			1.0
Never			0.95 (0.79, 1.14)
Former			1.08 (0.71, 1.66)
**Drinking**			
Current			1.0
Never			0.99 (0.86, 1.15)
Former			1.11 (0.81, 1.51)
**Medication use**			
No			1.0
Yes			3.1 (2.46, 4.57) ***
**NCDs**			
None			1.0
1			1.19 (1.03, 1.36) *
2			1.27 (1.05, 1.54) *
≥3			1.43 (1.13, 1.81) **
**Sleep time-mean (sd)**			0.86 (0.83, 0.89) ***

**ADL-mean (sd)**	0.97 (0.95, 0.99) **
**IADL-mean (sd)**	1.13 (1.10, 1.16) ***

**P*<0.05, ***P*<0.01, ****P*<0.001

OR: odds ratio, CI: confidence interval

Unadjusted model: fall accidents alone

Model 1: controls for gender, age, marital status, education level, personal annual income, residence, health insurance

Model 2: controls for Model 1 covariates, as well as self-rated health, self-rated eyesight, smoking, drinking, medication use, NCDs, sleep time, ADL, and IADL.

Table 5. Association between falls and depressive symptoms among 70-79 aged older adults in China, CLHLS 2018 (n = 2831)

Characteristics	Unadjusted model	Model 1	Model 2
	OR (95% CI)	OR (95% CI)	OR (95% CI)
**Falls**			
No	1.0	1.0	1.0
Yes	2.03 (1.70, 2.42) ***	1.82 (1.51, 2.18) ***	1.42 (1.16, 1.74) ***
**Sex**			
Female		1.0	1.0
Male		0.62 (0.52, 0.74) ***	0.77 (0.59, 0.99)
**Marital status**			
Others		1.0	1.0
Married		0.77 (0.64, 0.92) **	0.78 (0.64, 0.95) *
**Education**			
Illiterate		1.0	1.0
Primary		0.81 (0.68, 0.97) *	0.84 (0.69, 1.02)
Middle		0.6 (0.39, 0.91) *	0.67 (0.43, 1.04)
College or higher		0.26 (0.10, 0.68) **	0.28 (0.11, 0.76) *
**Annual income (CNY)**			
＜15000		1.0	1.0
15000-25000		0.79 (0.57, 1.11)	0.86(0.60, 1.23)
＞25000		0.62 (0.49, 0.80) ***	0.65(0.50, 0.85) **
**Residence**			
Rural		1.0	1.0
Urban		0.87 (0.69, 1.09) *	0.93 (0.73, 1.19)
**Health insurance**			
No		1.0	1.0
Yes		0.91 (0.61, 1.38)	0.87 (0.56, 1.37)
**Self-rated health**			
Good			1.0
Fair			0.53 (0.44, 0.65) ***
Bad			0.32 (0.24, 0.43) ***
**Self-rated eyesight**			
Good			1.0
Fair			0.73 (0.59, 0.91) **
Bad			0.88 (0.57, 1.34)
**Smoking**			
Current			1.0
Never			1.08 (0.85, 1.38)
Former			1.10 (0.63, 1.95)
**Drinking**			
Current			1.0
Never			0.95 (0.77, 1.18)
Former			1.45 (0.93, 2.26)
**Medication use**			
No			1.0
Yes			3.14(2.14, 4.79) ***
**NCDs**			
None			1.0
1			1.11 (0.91, 1.36)
2			1.36 (1.04, 1.79) *
≥3			1.52 (1.10, 2.10) *
**Sleep time-mean (sd)**			0.84 (0.81, 0.88) ***

**ADL-mean (sd)**	0.97 (0.93, 1.00)
**IADL-mean (sd)**	1.09 (1.06, 1.12) ***

**P*<0.05, ***P*<0.01, ****P*<0.001

OR: odds ratio, CI: confidence interval

Unadjusted model: fall accidents alone

Model 1: controls for gender, age, marital status, education level, personal annual income, residence, health insurance

Model 2: controls for Model 1 covariates, as well as self-rated health, self-rated eyesight, smoking, drinking, medication use, NCDs, sleep time, ADL, and IADL.

Table 6. Association between falls and depressive symptoms among 80- aged older adults in China, CLHLS 2018 (n = 701)

Characteristics	Unadjusted model	Model 1	Model 2
	OR (95% CI)	OR (95% CI)	OR (95% CI)
**Falls**			
No	1.0	1.0	1.0
Yes	1.46 (1.04, 2.04) *	1.38 (0.98, 1.94)	1.05 (0.72, 1.53)
**Sex**			
Female		1.0	1.0
Male		0.55 (0.38, 0.79) **	0.65 (0.4, 1.05)
**Marital status**			
Others		1.0	1.0
Married		1.1 (0.78, 1.54)	1.08 (0.75, 1.55)
**Education**			
Illiterate		1.0	1.0
Primary		0.90 (0.60, 1.35)	0.90 (0.59, 1.39)
Middle		0.54 (0.21, 1.42)	0.53 (0.19, 1.44)
College or higher		0.74 (0.27, 2.03)	0.87 (0.31, 2.50)
**Annual income (CNY)**			
＜15000		1.0	1.0
15000-25000		1.10 (0.52, 2.36)	1.04 (0.47, 2.29)
＞25000		0.76 (0.48, 1.21)	0.77 (0.47, 1.25)
**Residence**			
Rural		1.0	1.0
Urban		0.84 (0.55, 1.28)	0.90 (0.57, 1.41)
**Health insurance**			
No		1.0	1.0
Yes		0.99 (0.51, 1.93)	0.87 (0.43, 1.79)
**Self-rated health**			
Good			1.0
Fair			0.78 (0.52, 1.16)
Bad			0.50 (0.29, 0.86) *
**Self-rated eyesight**			
Good			1.0
Fair			0.76 (0.50, 1.15)
Bad			0.77 (0.31, 1.96)
**Smoking**			
Current			1.0
Never			1.06 (0.67, 1.70)
Former			0.85 (0.35, 2.09)
**Drinking**			
Current			1.0
Never			1.00 (0.66, 1.52)
Former			0.76 (0.30, 1.90)
**Medication use**			
No			1.0
Yes			3.02 (2.67, 5.72)
**NCDs**			
None			1.0
1			1.23 (0.82, 1.83)
2			1.93 (1.11, 3.37) *
≥3			2.2 (1.11, 4.35) *
**Sleep time-mean (sd)**			0.89 (0.83, 0.96) **

**ADL-mean (sd)**	1.04 (0.97, 1.11)
**IADL-mean (sd)**	1.06 (1.01, 1.11) *

**P*<0.05, ***P*<0.01, ****P*<0.001

OR: odds ratio, CI: confidence interval

Unadjusted model: fall accidents alone

Model 1: controls for gender, age, marital status, education level, personal annual income, residence, health insurance

Model 2: controls for Model 1 covariates, as well as self-rated health, self-rated eyesight, smoking, drinking, medication use, NCDs, sleep time, ADL, and IADL.

Table 7. Association between falls and depressive symptoms among urban older adults in China, CLHLS 2018 (n = 6964)

Characteristics	Unadjusted model	Model 1	Model 2
	OR (95% CI)	OR (95% CI)	OR (95% CI)
**Falls**			
No	1.0	1.0	1.0
Yes	1.71 (1.39, 2.12) ***	1.68 (1.35, 2.09) ***	1.22 (0.96, 1.56)
**Sex**			
Female		1.0	1.0
Male		0.77 (0.64, 0.94) **	0.82 (0.60, 1.12)
**Age**			
60-		1.0	1.0
70--		1.03 (0.83, 1.27)	0.87 (0.69, 1.09)
80-		1.14 (0.81, 1.60)	0.94 (0.64, 1.37)
**Marital status**			
Others		1.0	1.0
Married		0.85 (0.67, 1.07)	0.88 (0.69, 1.13)
**Education**			
Illiterate		1.0	1.0
Primary		0.72 (0.58, 0.89) **	0.81 (0.65, 1.03)
Middle		0.62 (0.46, 0.83) **	0.77 (0.56, 1.06)
College or higher		0.40 (0.22, 0.71) **	0.55 (0.30, 1.02)
**Annual income (CNY)**			
＜15000		1.0	1.0
15000-25000		0.90 (0.69, 1.18)	0.90 (0.67, 1.20)
＞25000		0.56 (0.45, 0.69) ***	0.60 (0.48, 0.76) ***
**Health insurance**			
No		1.0	1.0
Yes		0.77 (0.38, 1.57)	0.53 (0.25, 1.14)
**Self-rated health**			
Good			1.0
Fair			0.59 (0.47, 0.74) ***
Bad			0.36 (0.26, 0.51) ***
**Self-rated eyesight**			
Good			1.0
Fair			0.62 (0.49, 0.78) ***
Bad			0.75 (0.48, 1.17)
**Smoking**			
Current			1.0
Never			0.95 (0.71, 1.28)
Former			0.76 (0.44, 1.31)
**Drinking**			
Current			1.0
Never			1.07 (0.84, 1.37)
Former			1.33 (0.84, 2.10)
**Medication use**			
No			1.0
Yes			1.77 (0.51, 6.18)
**NCDs**			
None			1.0
1			1.49 (1.18, 1.87) **
2			1.41 (1.05, 1.91) *
≥3			1.55 (1.10, 2.17) *

**Sleep time-mean (sd)**	0.80 (0.75, 0.85) ***
**ADL-mean (sd)**	0.97 (0.94, 1.01)
**IADL-mean (sd)**	1.13 (1.09, 1.17) ***

**P*<0.05, ***P*<0.01, ****P*<0.001
OR: odds ratio, CI: confidence interval
Unadjusted model: fall accidents alone
Model 1: controls for gender, age, marital status, education level, personal annual income, residence, health insurance
Model 2: controls for Model 1 covariates, as well as self-rated health, self-rated eyesight, smoking, drinking, medication use, NCDs, sleep time, ADL, and IADL.

Table 8. Association between falls and depressive symptoms among rural older adults in China, CLHLS 2018 (n = 2575)

Characteristics	Unadjusted model	Model 1	Model 2
	OR (95% CI)	OR (95% CI)	OR (95% CI)
**Falls**			
No	1.0	1.0	1.0
Yes	1.99 (1.78, 2.23) ***	1.84 (1.63, 2.07) ***	1.42 (1.25, 1.61) ***
**Sex**			
Female		1.0	1.0
Male		0.59 (0.53, 0.66) ***	0.68 (0.57, 0.80) ***
**Age**			
60-		1.0	1.0
70--		0.95 (0.85, 1.07)	0.80 (0.71, 0.90) ***
80-		0.88 (0.72, 1.09)	0.71 (0.57,0.90) **
**Marital status**			
Others		1.0	1.0
Married		0.79 (0.69, 0.89) ***	0.80 (0.70, 0.92) **
**Education**			
Illiterate		1.0	1.0
Primary		0.79 (0.70, 0.89) ***	0.83 (0.74, 0.94) **
Middle		0.60 (0.46, 0.80) ***	0.68 (0.51, 0.91) **
College or higher		0.10 (0.01, 0.78) *	0.08 (0.01, 0.61) *
**Annual income (CNY)**			
＜15000		1.0	1.0
15000-25000		0.73 (0.59, 0.91) **	0.85 (0.68, 1.07)
＞25000		0.7 (0.59, 0.83) ***	0.79 (0.66, 0.94) **
**Health insurance**			
No		1.0	1.0
Yes		0.97 (0.74, 1.28)	0.96 (0.71, 1.29)
**Self-rated health**			
Good			1.0
Fair			0.53 (0.47, 0.60) ***
Bad			0.39 (0.33, 0.47) ***
**Self-rated eyesight**			
Good			1.0
Fair			0.81 (0.71, 0.92) **
Bad			0.65 (0.49, 0.86) **
**Smoking**			
Current			1.0
Never			1.01 (0.86, 1.18)
Former			1.20 (0.80, 1.79)
**Drinking**			
Current			1.0
Never			0.96 (0.84, 1.10)
Former			1.11 (0.83, 1.48)
**Medication use**			
No			1.0
Yes			3.87(2.77, 4.48) ***
**NCDs**			
None			1.0
1			1.09 (0.97, 1.24)
2			1.32 (1.11, 1.57) **
≥3			1.55 (1.24, 1.92) ***

**Sleep time-mean (sd)**	0.87 (0.85, 0.89) ***
**ADL-mean (sd)**	0.97 (0.95, 0.99) **
**IADL-mean (sd)**	1.10 (1.08, 1.12) ***

**P*<0.05, ***P*<0.01, ****P*<0.001

OR: odds ratio, CI: confidence interval

Unadjusted model: fall accidents alone

Model 1: controls for gender, age, marital status, education level, personal annual income, residence, health insurance

Model 2: controls for Model 1 covariates, as well as self-rated health, self-rated eyesight, smoking, drinking, medication use, NCDs, sleep time, ADL, and IADL.

Table 9. Association between falls and depressive symptoms among older adults with income＜15000 CNY in China, CLHLS 2018 (n = 6601)

Characteristics	Unadjusted model	Model 1	Model 2
	OR (95% CI)	OR (95% CI)	OR (95% CI)
**Falls**			
No	1.0	1.0	1.0
Yes	1.94 (1.73, 2.18) ***	1.80 (1.60, 2.03) ***	1.35 (1.19, 1.54) ***
**Sex**			
Female		1.0	1.0
Male		0.62 (0.56, 0.70) ***	0.68 (0.57, 0.81) ***
**Age**			
60-		1.0	1.0
70--		0.96 (0.85, 1.08)	0.82 (0.73, 0.93) **
80-		0.85 (0.69, 1.04)	0.71 (0.56, 0.89) **
**Marital status**			
Others		1.0	1.0
Married		0.78 (0.68, 0.88) ***	0.81(0.70, 0.92) **
**Education**			
Illiterate		1.0	1.0
Primary		0.78 (0.69, 0.87) ***	0.84 (0.74, 0.95) **
Middle		0.58 (0.43, 0.76) ***	0.64 (0.47, 0.86) **
College or higher		0.09 (0.01, 0.69) *	0.09 (0.01, 0.70) *
**Residence**			
Rural		1.0	1.0
Urban		0.87 (0.74, 1.01)	0.91 (0.78, 1.07)
**Health insurance**			
No		1.0	1.0
Yes		1.01 (0.76, 1.33)	0.94 (0.7, 1.27)
**Self-rated health**			
Good			1.0
Fair			0.55 (0.48, 0.62) ***
Bad			0.37 (0.31, 0.45) ***
**Self-rated eyesight**			
Good			1.0
Fair			0.79 (0.69, 0.90) ***
Bad			0.76 (0.58, 1.00)
**Smoking**			
Current			1.0
Never			1.01 (0.86, 1.19)
Former			1.23 (0.82, 1.83)
**Drinking**			
Current			1.0
Never			0.97 (0.84, 1.11)
Former			1.19 (0.89, 1.59)
**Medication use**			
No			1.0
Yes			3.11(2.58, 4.11) ***
**NCDs**			
None			1.0
1			1.14 (1.01, 1.3) *
2			1.29 (1.08, 1.53) **
≥3			1.61 (1.29, 2.01) ***
**Sleep time-mean (sd)**			0.86 (0.84, 0.89) ***

**ADL-mean (sd)**	0.98 (0.96, 1.00)
**IADL-mean (sd)**	1.10 (1.08, 1.12) ***

**P*<0.05, ***P*<0.01, ****P*<0.001
OR: odds ratio, CI: confidence interval
Unadjusted model: fall accidents alone
Model 1: controls for gender, age, marital status, education level, personal annual income, residence, health insurance
Model 2: controls for Model 1 covariates, as well as self-rated health, self-rated eyesight, smoking, drinking, medication use, NCDs, sleep time, ADL, and IADL.

Table 10. Association between falls and depressive symptoms among older adults with income of 15000-25000 CNY in China, CLHLS 2018 (n = 795)

Characteristics	Unadjusted model	Model 1	Model 2
	OR (95% CI)	OR (95% CI)	OR (95% CI)
**Falls**			
No	1.0	1.0	1.0
Yes	1.80 (1.23, 2.65) **	1.74 (1.17, 2.59) **	1.52 (0.98, 2.36)
**Sex**			
Female		1.0	1.0
Male		0.65 (0.46, 0.92) *	0.71 (0.41, 1.21)
**Age**			
60-		1.0	1.0
70--		1.06 (0.73, 1.54)	0.84 (0.56, 1.26)
80-		1.38 (0.65, 2.93)	1.11 (0.49, 2.49)
**Marital status**			
Others		1.0	1.0
Married		1.01 (0.66, 1.54)	1.04 (0.66, 1.66)
**Education**			
Illiterate		1.0	1.0
Primary		0.80 (0.57, 1.13)	0.77 (0.53, 1.11)
Middle		0.84 (0.46, 1.53)	0.77 (0.40, 1.47)
College or higher		0.77 (0.67, 1.04)	0.61 (0.57, 0.88)
**Residence**			
Rural		1.0	1.0
Urban		1.06 (0.75, 1.48)	0.97 (0.67, 1.41)
**Health insurance**			
No		1.0	1.0
Yes		0.39 (0.14,1.1)	0.44 (0.13, 1.51)
**Self-rated health**			
Good			1.0
Fair			0.7 (0.47, 1.05)
Bad			0.74 (0.42, 1.29)
**Self-rated eyesight**			
Good			1.0
Fair			0.42 (0.27, 0.65) ***
Bad			0.44 (0.19, 1.01)
**Smoking**			
Current			1.0
Never			0.93 (0.57, 1.51)
Former			0.65 (0.22, 1.95)
**Drinking**			
Current			1.0
Never			1.03 (0.67, 1.56)
Former			2.22 (1.01, 4.87) *
**Medication use**			
No			1.0
Yes			3.84 (2.21, 5.15) *
**NCDs**			
None			1.0
1			1.47 (0.98, 2.21)
2			1.43 (0.83, 2.47)
≥3			1.56 (0.84, 2.9)
**Sleep time-mean (sd)**			0.74 (0.66, 0.82) ***
**ADL-mean (sd)**			0.99 (0.92, 1.06)

**IADL-mean (sd)**	1.07 (0.99, 1.15)

*P<0.05, **P<0.01, ***P<0.001

OR: odds ratio, CI: confidence interval

Unadjusted model: fall accidents alone

Model 1: controls for gender, age, marital status, education level, personal annual income, residence, health insurance

Model 2: controls for Model 1 covariates, as well as self-rated health, self-rated eyesight, smoking, drinking, medication use, NCDs, sleep time, ADL, and IADL.

Table 11. Association between falls and depressive symptoms among older adults with income ＞25000 CNY in China, CLHLS 2018 (n = 2143)

Characteristics	Unadjusted model	Model 1	Model 2
	OR (95% CI)	OR (95% CI)	OR (95% CI)
**Falls**			
No	1.0	1.0	1.0
Yes	1.87 (1.47, 2.38) ***	1.83 (1.43, 2.34) ***	1.42 (1.09, 1.85) **
**Sex**			
Female		1.0	1.0
Male		0.65 (0.52, 0.82) ***	0.80 (0.56, 1.15)
**Age**			
60-		1.0	1.0
70--		0.98 (0.77, 1.24)	0.73 (0.56, 0.95) *
80-		1.20 (0.83, 1.74)	0.82 (0.54, 1.25)
**Marital status**			
Others		1.0	1.0
Married		0.85 (0.63, 1.15)	0.83 (0.60, 1.13)
**Education**			
Illiterate		1.0	1.0
Primary		0.70 (0.54, 0.91) **	0.74 (0.56, 0.99) **
Middle		0.59 (0.42, 0.83) **	0.72 (0.50, 1.03)
College or higher		0.42 (0.23, 0.76) **	0.52 (0.28, 0.98) *
**Residence**			
Rural		1.0	1.0
Urban		0.72 (0.57, 0.91) **	0.75 (0.59, 0.96) *
**Health insurance**			
No		1.0	1.0
Yes		0.97 (0.43, 2.21)	1.01 (0.41, 2.48)
**Self-rated health**			
Good			1.0
Fair			0.51 (0.39, 0.66) ***
Bad			0.35 (0.24, 0.51) ***
**Self-rated eyesight**			
Good			1.0
Fair			0.79 (0.60, 1.02)
Bad			0.53 (0.30, 0.92) *
**Smoking**			
Current			1.0
Never			0.97 (0.69, 1.35)
Former			0.97 (0.52, 1.81)
**Drinking**			
Current			1.0
Never			1.07 (0.82, 1.38)
Former			0.80 (0.44, 1.45)
**Medication use**			
No			1.0
Yes			2.43 (0.62, 4.62)
**NCDs**			
None			1.0
1			1.23 (0.94, 1.60)
2			1.53 (1.08, 2.15) *
≥3			1.30 (0.87, 1.94)
**Sleep time-mean (sd)**			0.87 (0.82, 0.93) ***

**ADL-mean (sd)**	0.93 (0.89, 0.98) **
**IADL-mean (sd)**	1.15 (1.10, 1.20) ***

*P<0.05, **P<0.01, ***P<0.001

OR: odds ratio, CI: confidence interval

Unadjusted model: fall accidents alone

Model 1: controls for gender, age, marital status, education level, personal annual income, residence, health insurance

Model 2: controls for Model 1 covariates, as well as self-rated health, self-rated eyesight, smoking, drinking, medication use, NCDs, sleep time, ADL, and IADL.
